# Supplementary figures and images for: Genomic locus proteomic screening identifies the NF-κB signaling pathway components NFκB1 and IKBKG as transcriptional regulators of Ripk3 in endothelial cells
Source: PLoS One. 2021 Jun 21;16(6):e0253519. doi: 10.1371/journal.pone.0253519 (PMC8216549; doi:10.1371/journal.pone.0253519)

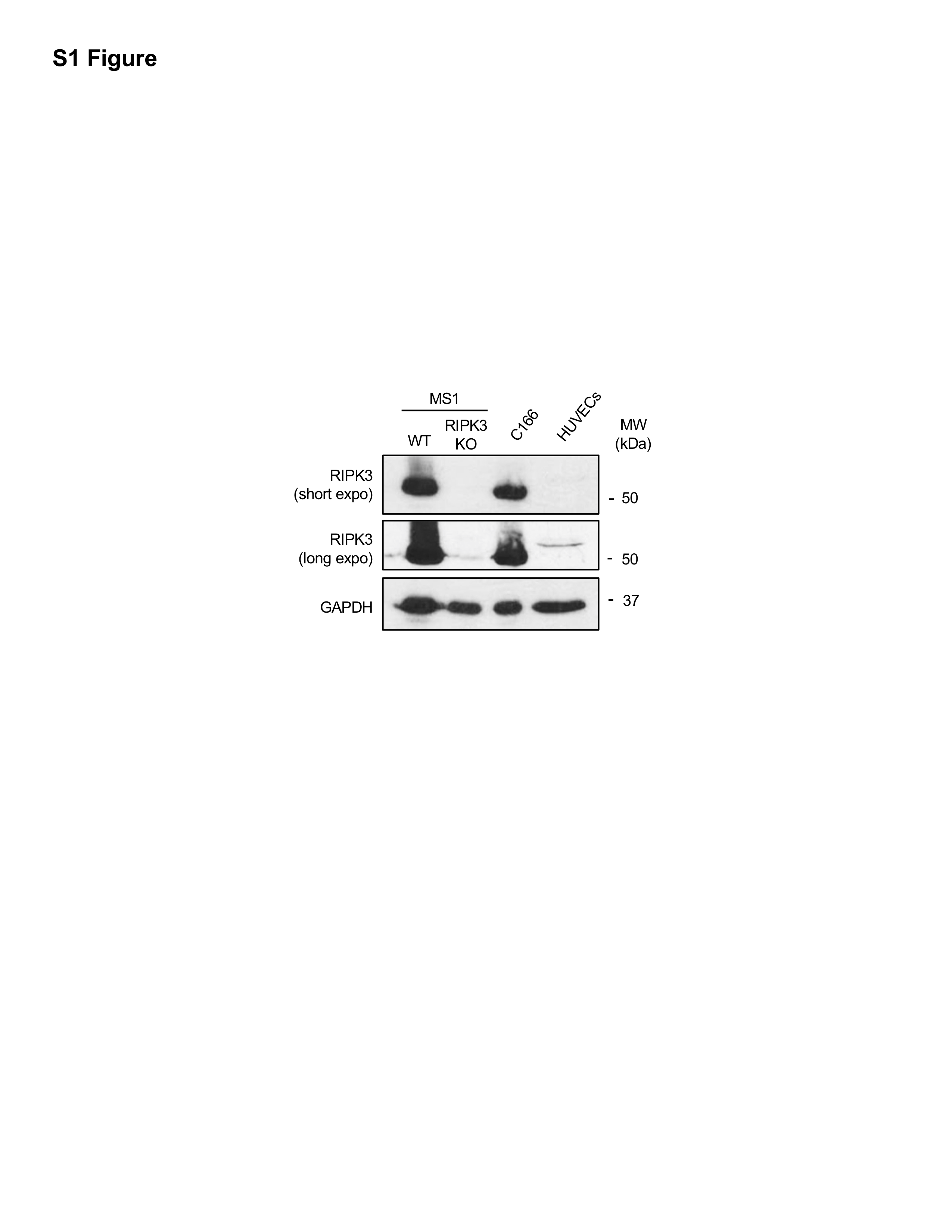

Supplement: S1 Fig — RIPK3 protein levels were analyzed by immunoblotting in immortalized MS1 ECs (adult murine pancreas-derived), immortalized C166 ECs (embryonic murine yolk sac-derived), and primary human umbilical vein endothelial cells (HUVECs). MS1 RIPK3 knockout (KO) ECs were generated by CRISPR/Cas9 technology and were included as a negative control. Note that the molecular weight (MW) of human RIPK3 is slightly greater than that of mouse RIPK3. (TIF) [file pone.0253519.s001.tif]

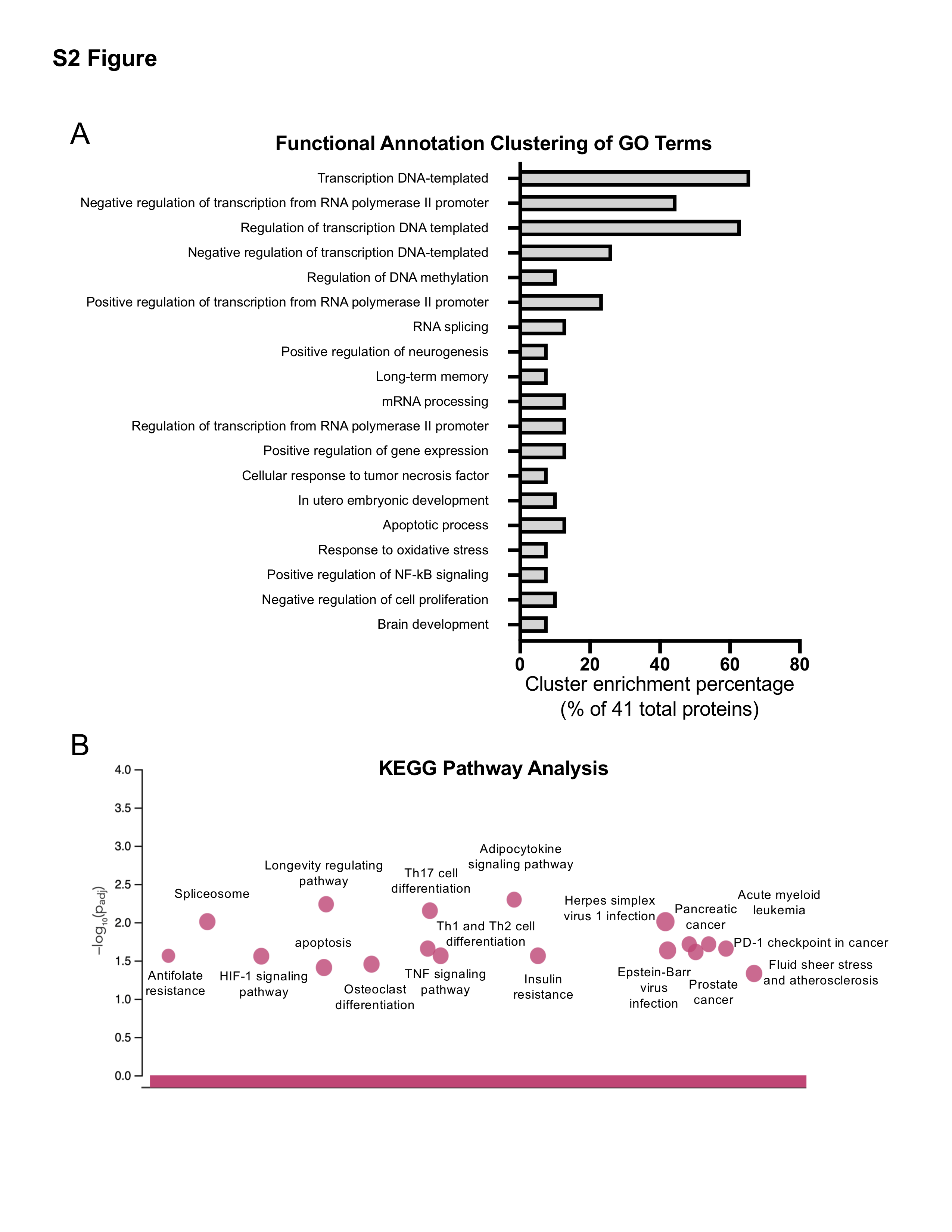

Supplement: S2 Fig — (A) Functional annotation clustering of GO terms associated with the 41 filtered Ripk3 GLoPro proteins shown in Fig 1C by DAVID. (B) Kyoto Encyclopedia of Genes and Genomes (KEGG) pathway analysis of the 41 filtered Ripk3 GLoPro proteins shown in Fig 1C. This analysis was performed using g:Profiler and included a false discovery rate of p<0.05 after Benjamini-Hochberg correction for multiple comparisons. (TIF) [file pone.0253519.s002.tif]

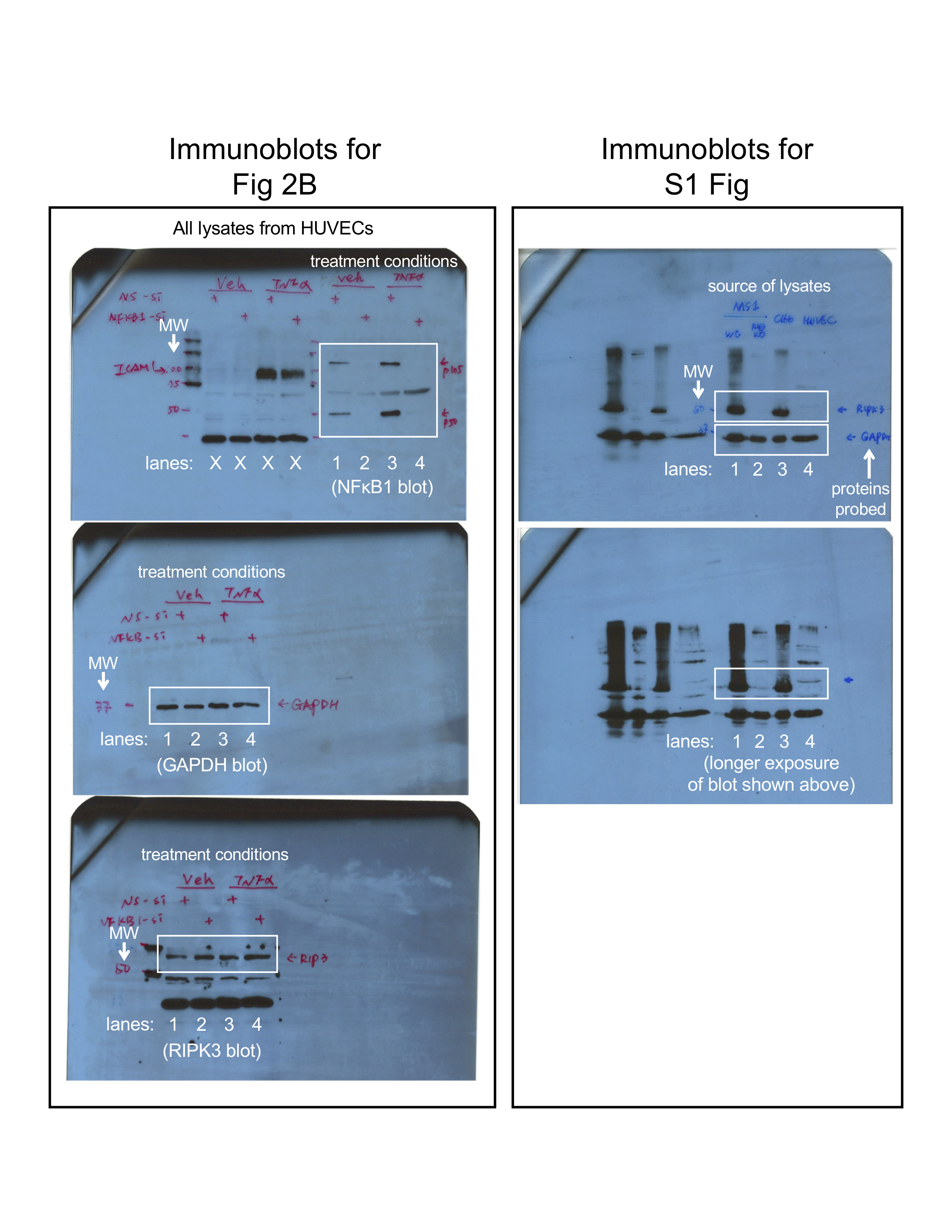

Supplement: S1 Raw images — (TIF) [file pone.0253519.s012.tif]
