# Supplementary material for: Genomic locus proteomic screening identifies the NF-κB signaling pathway components NFκB1 and IKBKG as transcriptional regulators of Ripk3 in endothelial cells
Source: PLoS One. 2021 Jun 21;16(6):e0253519. doi: 10.1371/journal.pone.0253519 (PMC8216549; doi:10.1371/journal.pone.0253519)
Supplement: S5 Table — (DOCX) [file pone.0253519.s007.docx]

**S5 Table. Related to Materials and Methods; Key resources used in this study**

| **REAGENT or RESOURCE** | **SOURCE** | **IDENTIFIER** |
| --- | --- | --- |
| Antibodies | | |
| Rabbit polyclonal anti-NFκB1 p105/p50 | Cell Signaling Technology | Cat #: 3035S  RRID: AB_330564 |
| Rabbit polyclonal anti-IgG | Millipore | Cat #: NI01-100UG Lot 3050723  RRID: AB_10681285 |
| IKBKG Antibody | Abcam | Cat# 178872  RRID: AB_2847887 |
| RIPK3 | Novus Biology | Cat #: NBP1-77299  RRID: AB_11040928 |
| GAPDH | Sigma-Aldrich | Cat #: G9545  RRID: AB_796208 |
| Goat anti-Rabbit IgG HRP | Sigma-Aldrich | Cat #: A6667  RRID: AB_258307 |
| Bacterial and Virus Strains | | |
| *E.coli* DH5α | Thermo Fisher | Cat #: 18258012 |
| Stable Competent *E. coli* | New England BioLabs | Cat #: C3040I |
| Chemicals, Peptides, and Recombinant Proteins | | |
| Doxycycline | Cayman Chemical | Cat #: 14422 |
| Puromycin | Cayman Chemical | Cat #: 13884 |
| Hygromycin B | Cayman Chemical | Cat #: 14291 |
| Biotin tyramide phenol | APExBIO | Cat #: A8011 |
| Protease inhibitor cocktail | Thermo Fisher | Cat #: 8340 |
| Streptavidin magnetic beads | New England BioLabs | Cat #: S1420S |
| Protein A/G-conjugated agarose beads | Millipore | Cat #: IP05 |
| Z-VAD-FMK | Selleckchem | Cat #: S7023 |
| Necrosulfonamide | Selleckchem | Cat #: S8251 |
| Propidium iodide | BD Pharmingen | Cat #: 556463 |
| TNFα | R&D Systems | Cat#: 410-MT |
| Critical Commercial Assays | | |
| RNeasy mini kit | QIAGEN | Cat #: 74106 |
| Maxi Prep kit | QIAGEN | Cat #: 12163 |
| iSCRIPT™ Reverse Transcriptase Kit | Bio-Rad | Cat #: 1708891 |
| 2X SYBR green qPCR master mix | Applied Biosystems | Cat #: 4312704 |
| ChIP-IT® Express Kit | Active Motif | Cat #: 53008 |
| Deposited Data | | |
| ProteomeXchange Consortium via the PRIDE partner repository | https://www.ebi.ac.uk/pride/ | PXD025675 |
| Experimental Models: Cell Lines/primary cells | | |
| MS1 (MILE SVEN 1) endothelial cells | ATCC | Cat #: CRL-2279  RRID: CVCL_6502 |
| C166 endothelial cells | ATCC | Cat #: CRL-2581  RRID: CVCL_6581 |
| Primary HUVECs | ATCC | Cat #: PCS-100-010  RRID: N/A |
| HEK293T cells | ATCC | Cat #: CRL-3216  RRID: CVCL_0063 |
| Oligonucleotides | | |
| Primers for cloning, see S6 Table | This paper | N/A |
| Primers for qRT-PCR, see S7 Table | This paper | N/A |
| Primers for ChIP-qPCR, see S8 Table | This paper | N/A |
| Recombinant DNA | | |
| Information about all plasmids, See S9 Table | This paper | See Table S9 |
| Software and Algorithms | | |
| Prism 8 | GraphPad | https://www.graphpad.com/scientific-software/prism/ |
| GPP Web Portal | Broad Institute | https://portals.broadinstitute.org/gpp/public/ |
| CHOPCHOP | (Labun et al., 2019) | https://chopchop.cbu.uib.no |
| Block-iT RNAi Designer | Thermo Fisher | https://rnaidesigner.thermofisher.com/rnaiexpress/ |
| Perseus v1.6.12.0 | MaxQuant (Max Planck Institute) | https://maxquant.net/perseus/ |
| DAVID Bioinformatics Database v6.8 | Laboratory of Immunopathogenesis and Bioinformatics | https://david.ncifcrf.gov/tools.jsp |
| g:profiler | Institute of Computer Science, University of Tartu | http://biit.cs.ut.ee/gprofiler/gost |
| MaxQuant v1.6.2.10 | Max Planck Institute | https://www.maxquant.org |
