# Supplementary material for: Genomic locus proteomic screening identifies the NF-κB signaling pathway components NFκB1 and IKBKG as transcriptional regulators of Ripk3 in endothelial cells
Source: PLoS One. 2021 Jun 21;16(6):e0253519. doi: 10.1371/journal.pone.0253519 (PMC8216549; doi:10.1371/journal.pone.0253519)
Supplement: S6 Table — (DOCX) [file pone.0253519.s008.docx]

**S6 Table. Related to Materials and Methods;** **Cloning primers used in this study**

**Cloning Primers**

| **Name** | **(5' to 3')** |
| --- | --- |
| **Construction of GLoPro sgRNA** |  |
| NT-sgRNA_F | ACACCGTATTACTGATATTGGTGGGG |
| NT-sgRNA_R | AAAACCCCACCAATATCAGTAATACG |
| 261mRipk3-gRNA_F | ACACCGCGGAGTAACGCTTCTAGTAAG |
| 261mRipk3-gRNA_R | AAAACTTACTAGAAGCGTTACTCCGCG |
| 115mRipk3-gRNA_F | ACACCGGGAGTCAATCGTTCCTGGAG |
| 115mRipk3-gRNA_R | AAAACTCCAGGAACGATTGACTCCCG |
| 39mRipk3-gRNA_F | ACACCGTCGCTCATTCAAAGTCCGGG |
| 39mRipk3-gRNA_R | AAAACCCGGACTTTGAATGAGCGACG |
|  |  |
|  |  |
| **Construction of RIPK3 KO sgRNA** |  |
| mRipk3- CRISPR KO_Ex9_F | CACCGTACCTGTCATTGGATTCGGT |
| mRipk3- CRISPR KO_Ex9_R | AAACACCGAATCCAATGACAGGTAC |
|  |  |
