# Supplementary material for: Genomic locus proteomic screening identifies the NF-κB signaling pathway components NFκB1 and IKBKG as transcriptional regulators of Ripk3 in endothelial cells
Source: PLoS One. 2021 Jun 21;16(6):e0253519. doi: 10.1371/journal.pone.0253519 (PMC8216549; doi:10.1371/journal.pone.0253519)
Supplement: S7 Table — (DOCX) [file pone.0253519.s009.docx]

**S7 Table. Related to Materials and Methods;** **qRT-PCR primers used in this study**

**qRT-PCR primers (Human)**

| **Gene Name** | **Forward (5' to 3')** | **Reverse (5' to 3')** |
| --- | --- | --- |
| *Ripk3* | GAGACTCCCGGCTTAGAAGG | TCCTTTACCGTGGAGACAGC |
| *Actb* | CTCTTCCAGCCTTCCTTCCT | AGCACTGTGTTGGCGTACAG |
| *Gapdh* | GAGTCAACGGATTTGGTCGT | GACAAGCTTCCCGTTCTCAG |
| *Rn18s* | CCCGAAGCGTTTACTTTGAAA | CGCGGTCCTATTCCATTATTC |
|  |  |  |
