# Supplementary material for: Genomic locus proteomic screening identifies the NF-κB signaling pathway components NFκB1 and IKBKG as transcriptional regulators of Ripk3 in endothelial cells
Source: PLoS One. 2021 Jun 21;16(6):e0253519. doi: 10.1371/journal.pone.0253519 (PMC8216549; doi:10.1371/journal.pone.0253519)
Supplement: S8 Table — (DOCX) [file pone.0253519.s010.docx]

**S8 Table. Related to Materials and Methods;** **ChIP-qPCR primers used in this study**

**ChIP-qPCR primers (Human)**

| **Distance from *Ripk3* TSS** | **Forward (5' to 3')** | **Reverse (5' to 3')** |
| --- | --- | --- |
| -0.2 kb | CAGGAAATCCTGTGGGAAAA | TCCTTGACTAGCGTTTCCTGA |
| -0.5 kb | AGCCCTGAAGCCTATGTGAA | CGAACCCTAAGGGGATAGGT |
| -1.0 kb | TTCCCACAACCTCCAGTCAT | GCACATGCTCTGGACACAGT |
| -14 kb | TCCTCCCTCTCACCTTACCT | AGGGTGGCATGAGTGAATGA |
|  |  |  |
