# Supplementary material for: Genomic locus proteomic screening identifies the NF-κB signaling pathway components NFκB1 and IKBKG as transcriptional regulators of Ripk3 in endothelial cells
Source: PLoS One. 2021 Jun 21;16(6):e0253519. doi: 10.1371/journal.pone.0253519 (PMC8216549; doi:10.1371/journal.pone.0253519)
Supplement: S9 Table — (DOCX) [file pone.0253519.s011.docx]

**S9 Table: Related to Materials and Methods;** **Plasmids used in this study**

| **Plasmid** | **Parent vector** | **Primer set/**  **Restriction enzymes used for cloning** | **Reference/Supplier** |
| --- | --- | --- | --- |
| iCaspex | - | - | (Myers et al., 2018)  Addgene Plasmid  Cat #: 97421  RRID: Addgene_97421 |
| pLenti SpBsmBI sgRNA Hygro | - | - | (Pham et al., 2016)  Addgene Plasmid  Cat #: 62205  RRID: Addgene_62205 |
| NT-gRNA | pLenti SpBsmBI sgRNA Hygro | NT-sgRNA-F  NT-sgRNA-R  /BsmBI | This paper |
| g261 | pLenti SpBsmBI sgRNA Hygro | 261mRipk3-sgRNA-F  261mRipk3-sgRNA-R  /BsmBI | This paper |
| g115 | pLenti SpBsmBI sgRNA Hygro | 115mRipk3-sgRNA-F  115mRipk3-sgRNA-R  /BsmBI | This paper |
| g39 | pLenti SpBsmBI sgRNA Hygro | 39mRipk3-sgRNA-F  39mRipk3-sgRNA-R  /BsmBI | This paper |
| pSpCas9 (BB)-2A-GFP |  |  | (Ran et al., 2013)  Cat #: 48138  RRID: Addgene_48138 |
| psPAX2 | - | - | Gift from Dr. Lijun Xia’s lab (OMRF)  RRID: Addgene_12260 |
| pMD2.G | - | - | Gift from Lijun Xia’s lab (OMRF)  RRID: Addgene_12259 |
