# Supplementary material for: Genomic locus proteomic screening identifies the NF-κB signaling pathway components NFκB1 and IKBKG as transcriptional regulators of Ripk3 in endothelial cells
Source: PLoS One. 2021 Jun 21;16(6):e0253519. doi: 10.1371/journal.pone.0253519 (PMC8216549; doi:10.1371/journal.pone.0253519)
Supplement: S1 References — (DOCX) [file pone.0253519.s013.docx]

**Supplemental References**

Labun, K., Montague, T.G., Krause, M., Torres Cleuren, Y.N., Tjeldnes, H., and Valen, E. (2019). CHOPCHOP v3: expanding the CRISPR web toolbox beyond genome editing. Nucleic Acids Res *47*, W171-W174.

Myers, S.A., Wright, J., Peckner, R., Kalish, B.T., Zhang, F., and Carr, S.A. (2018). Discovery of proteins associated with a predefined genomic locus via dCas9-APEX-mediated proximity labeling. Nat Methods *15*, 437-439.

Pham, H., Kearns, N.A., and Maehr, R. (2016). Transcriptional Regulation with CRISPR/Cas9 Effectors in Mammalian Cells. Methods Mol Biol *1358*, 43-57.

Ran, F.A., Hsu, P.D., Wright, J., Agarwala, V., Scott, D.A., and Zhang, F. (2013). Genome engineering using the CRISPR-Cas9 system. Nat Protoc *8*, 2281-2308.
