# Supplementary material for: Genomic locus proteomic screening identifies the NF-κB signaling pathway components NFκB1 and IKBKG as transcriptional regulators of Ripk3 in endothelial cells
Source: PLoS One. 2021 Jun 21;16(6):e0253519. doi: 10.1371/journal.pone.0253519 (PMC8216549; doi:10.1371/journal.pone.0253519)
Supplement: Attachment — Submitted filename: Response to Reviewers PONE-D-2-35540.docx [file pone.0253519.s014.docx]

**Courtney T. Griffin, Ph.D.**

*Member, Cardiovascular Biology Research Program*

*Scott Zarrow Chair in Biomedical Research*

*Scientific Director, Oklahoma Center for Adult Stem Cell Research*

*President-elect, North American Vascular Biology Organization*

Oklahoma Medical Research Foundation

825 Northeast 13^th^ Street

Oklahoma City, OK 73104-5046

Telephone: 405-271-7073

Fax: 405-271-3137

Email: Courtney-Griffin@omrf.org

April 28, 2021

Ramani Ramchandran

Academic Editor

PLOS ONE

RE: Resubmission of PONE-D-20-35540

Dear Dr. Ramchandran:

Thank you for handling the recent reviews of our manuscript “Genomic locus proteomic screening identifies NFκB1 as a transcriptional regulator of *Ripk3* in endothelial cells.” We appreciated the Reviewers’ constructive critiques and have generated new data and re-written/organized sections of our original manuscript to address their queries, as detailed below.

**Reviewer #1**

1. *“RIPK3 is predominantly in the cytoplasm, by any means did the author assess the interaction is cytoplasmic or nuclear.”*

We agree with the Reviewer that RIPK3 is predominantly a cytoplasmic protein in cell types that have been assessed for its localization. Because the goal of this manuscript was to identify transcriptional regulators of *Ripk3*, the genomic locus proteomic (GLoPro) screen that we utilized here was performed to identify proteins that interact with the *Ripk3* promoter—not with RIPK3 protein itself. Nevertheless, we recently performed an unbiased proteomic screen to identify RIPK3-interacting proteins in both HUVECs and the MS1 endothelial cell line [1]. NFκB1 and IKBKG (newly analyzed in this revision) did not emerge in our mass spectrometry analysis of proteins that immunoprecipitated with RIPK3 in these endothelial cell lines. Therefore, our collective data indicate that NFκB1 and IKBKG interact with the *Ripk3* promoter in endothelial cells but not with RIPK3 protein itself.

2. *“It is more concerning that if NFκB1 knockdown caused significant cell death in HUVECs grown under basal conditions or with TNFα stimulation, when was the transcriptional and translational experiments done?”*

RIPK3 transcriptional and translational experiments (original Figures 5A and 5B; revised Figures 2A and 2B, respectively) were performed at 48 hr (24 hr after NFκB1 knockdown plus 24 hr after additional vehicle or TNFα treatment). This is comparable to the timecourse of analysis for cell death (original Figures 5C and 5D; revised Figures 2C and 2D). Please note that we routinely rinse cultured cells before all analyses to remove dead cells and debris. Moreover, transcription is evaluated by qPCR using three housekeeping genes as internal controls, and GAPDH is used as a loading control for our western blots. Therefore, we are confident that we are measuring *Ripk3* transcripts and protein in live cells rather than dead ones.

3. *“It implies that TNFα only have transcriptional regulation of RIPK3 but not translational.”*

TNFα treatment has a small impact on *Ripk3* transcription (original Figure 5A; revised Figure 2A, lanes 1 vs. 3: *p*=0.28) and translation (original Figure 5B; revised Figure 2A, lanes 1 vs. 3), but a bigger impact when combined with NFκB1 knockdown (original Figure 5A and 5B; revised Figures 2A and 2B, lanes 1 vs. 4). Admittedly, the transcriptional impact of this combination is greater than the translational impact (8.2-fold transcriptional change vs. 2.6-fold translational change). Nevertheless, the functional relevance of TNFα treatment plus NFκB1 knockdown is evident from the cell death assays (original Figure 5C; revised Figure 2C, lane 6) (original Figure 5D; revised Figure 2D, lane 4).

4. *“What is the rationale for using pan-caspase inhibitor Z-VAD-FMK to rescue NFκB1* *knockdown mediated cell death in HUVECS? It appears too vague.”*

Z-VAD-FMK is a pan-caspase inhibitor that has been used since the mid-1990s to block programmed cell death in a variety of mammalian cell lines [2]. It is commonly employed to distinguish apoptosis from non-caspase-mediated forms of cell death such as necroptosis [3]. Z-VAD-FMK is also frequently used in combination with TNFα stimulation to block apoptosis and drive cultured cells toward RIPK3-dependent necroptosis [4, 5]. When we determined that the elevated cell death we detected in HUVECs treated with TNFα and NFκB1 knockdown was RIPK3-dependent (original Figure 5C; revised Figure 2C, lanes 6 vs. 8), we assumed the death would be mechanistically necroptotic rather than apoptotic. Therefore, we predicted that we would be able to block the cell death with the necroptosis inhibitor necrosulfonamide (NSA) but not with the apoptosis inhibitor Z-VAD-FMK. However, our cell death blocking experiments gave the opposite results (original Figure 5D; revised Figure 2D), indicating that the RIPK3-mediated death we stimulated in HUVECs was apoptotic and/or caspase-dependent rather than necroptotic. This result adds to our lab’s growing evidence that RIPK3-mediated necroptosis is difficult to stimulate or detect in endothelial cells both in vitro and in vivo [6, 7].

5. *“If endothelial RIPK3 expression must be tightly regulated to maintain vascular integrity and homeostasis at different stages of life, the low-level expression of RIPK3 in HUVECs suggest HUVECs may not be the right model. Could you please clarify the rationale for using HUVECs?”*

Although the immortalized MS1 endothelial cell line we used for the GLoPro studies was critical for generating the large number of cells required for this assay, we believe that HUVECs are more appropriate for functional validation studies since they are primary cells and are maintained at low passage number (endothelial cells are notoriously plastic and prone to de-differentiation in prolonged culture[8]). Notably, HUVECs are responsive to TNFα-mediated NF-κB activation and downstream signaling events [9], which makes them particularly relevant to this study. In addition, we recently used HUVECs to study novel angiogenic roles for RIPK3, and they faithfully recapitulated endothelial cell behaviors that occurred upon *Ripk3* genetic deletion in vivo [1]. Therefore, although RIPK3 is expressed at relatively low levels in unstimulated HUVECs compared to immortalized endothelial cells (see Figure S1), RIPK3 is clearly functional and biologically relevant in HUVECs and requires regulated maintenance.

6. *“In the discussion section, many studies have focused on how post-translational modifications impact RIPK3 stability, particularly in the context of immune cells, could you please give any references to that.”*

We have added relevant references, as requested (see new references #34, 35). Please see also references #13-16, which were previously cited in our Introduction.

7. *“In the discussion section, according to the author, the study was designed to identify regulators such as RIPK3 in ECs, is HUVEC the right model to study such kind of regulator. The rationale is not clear.”*

Please see our response to #5 above.

8. *“In the discussion section some of the information about NF-κB* *can be moved to introduction.”*

We have moved background information about NF-κB signaling in ECs to the introduction, as suggested.

**Reviewer #2**

1. *“The results section is poorly written and has not explained in depth to support their experimental conclusions.”*

We have revised the Results section to clarify the techniques and motivations behind all the experiments.

2. *“Figure 2 need better color contrast to observe heat map matrix. Secondly, merge Figure 1 and 2.”*

We have revised and merged Figures 1 and 2, as suggested.

3. *“Figure 3 should be moved to Supplementary. It does not add any experimental parameters for the manuscript.”*

The original Figure 3 is now Figure S2, as suggested.

4. *“GLoPro-CRISPR approach/screening is the backbone of the manuscript. Authors should validate more identified proteins as are listed in Figure 2.”*

We appreciated this important recommendation and have now evaluated IKBKG (also known as NEMO or IKK-γ) for its ability to bind the *Ripk3* promoter and regulate its transcription. We chose this protein among those gleaned from the GLoPro screen because of its important role in the NF-κB signaling pathway, which is a critical component of our manuscript. Notably, IKBKG is best known for its cytoplasmic roles in activating the NF-κB pathway and in protecting cells from TNFα-induced apoptosis [10, 11]. However, IKBKG can also undergo nuclear translocation upon stress induction in a pre-B cell line [12]. Interestingly, we now show that IKBKG is primarily expressed in the nuclei of HUVECs grown under basal conditions, and its nuclear expression appears to be further elevated upon TNFα treatment (see revised Figure 3A). Importantly, knockdown of IKBKG significantly elevates *Ripk3* transcription in TNFα-treated HUVECs (see revised Figure 3B, lane 4)—similarly to the *Ripk3* elevation seen with NFκB1 knockdown plus TNFα treatment (revised Figure 2A, lane 4)—which indicates that both of these NF-κB pathway components suppress endothelial *Ripk3* transcription upon TNFα treatment. Importantly, our ability to ChIP IKBKG to the *Ripk3* promoter in HUVECs grown under basal conditions (revised Figure 4A, lane 4) and to detect its recruitment to the -0.2 kb and -1.0 kb sites of the promoter after TNFα treatment (revised Figure 4B, lanes 2 and 6) support our conclusion that IKBKG directly regulates *Ripk3* transcription in HUVECs, particularly after TNFα treatment. These new ChIP, immunostaining, and transcriptional data further validate our GLoPro approach while also providing novel insights into nuclear roles for IKBKG in endothelial cells.

5. *“Supporting Figure S1 show that HUVEC cells have very low protein expression (~negligible) of RIPK3 as compared to MS1 cells, however, ChIP assay, lane NFκB1 in Figure 4 panel A & B, do not co-relate. It is very important to have quantitative enrichment or fold change for each test samples with some Statistically Analysis.”*

To address this important request, we repeated all our HUVEC ChIP analyses in triplicate and have analyzed the data by qPCR for quantitative analysis of NFκB1 and IKBKG enrichment at the *Ripk3* promoter (see revised Figure 4). Also, please see responses to Reviewer 1 (#3 and #5) about *Ripk3* transcript vs. protein levels in HUVECs

6. *“Figure 5 panel B need quantification as well as more detail in result section. Why NFkB1 (p105/p50) is high? What are other bands at MW 105 and 50?”*

The original Figure 5B is now revised Figure 2B. Please note that quantification (relative densitometry of RIPK3) is provided below the RIPK3 blot. For the NFκB1 blot, the antibody used recognizes both subunits of the protein (p105 and p50), which are shown on the blot at their corresponding molecular weights. We have now added arrows to clarify that those are the bands of interest (the band located between 105 and 50 kDa is a background band picked up by the antibody).

**Reviewer #3**

1. *(Major) “What is the resolution of GLoPro? Would you anticipate that all three probes would identify the same interacting proteins? For the MS1 NFκB1 ChIP data, it seems to only interact with a distal region (-1 kb), but the GLoPro showed interaction with all three probes, which were more proximal to the promoter. Is this due to differences in resolution between these two techniques?”*

Myers et al reported that the GLoPro technique biotinylates proteins within 400bp of a genomic target [13]. Therefore, given that our sgRNAs spanned a region of 300 bp (-261 through +39 of the *Ripk3* promoter), we do predict that they would identify a largely overlapping set of interacting proteins. Our original ChIP for NFκB1 binding in MS1 cells showed the protein sitting ~1 kb upstream (-1.0 kb) of the *Ripk3* transcription start site under basal conditions (original Figure 4A, also shown in Figure R1D below). For this revision we performed more extensive ChIP analysis of HUVECs and found NFκB1 and IKBKG sitting ~0.5 kb upstream of the TSS under basal conditions (*p*=0.14 and 0.03, respectively; see revised Figure 4A). Because the majority of our sonication fragments for ChIP are ~250-750 bp in size, we believe our ChIP and GLoPro data are consistent with each other in terms of indicating that NFκB1 and IKBKG can bind within 1 kb of the *Ripk3* TSS in endothelial cells.

2. *(Major) “Are there cis elements in the RIPK3 promoter that match the transcription factors that were identified? This is especially relevant for NFκB1. Where are the NF-κB elements and is the binding predicted to be conserved across species? Does mutation of NF-κB motifs affect promoter activity in a luciferase assay?”*

We used the Meme Suite (<https://meme-suite.org/meme/>) to analyze the murine *Ripk3* promoter for predicted NF-κB transcription factor binding sites. The position weight matrix shown below in Figure R1 demonstrates an NF-κB consensus binding motif located between -1258 bp and -1237 bp within the *Ripk3* promoter. This binding motif is based on the described consensus DNA sequence of 5’-GGGRNYYYCC-3’ (in which R is a purine, Y is a pyrimidine, and N is any nucleotide) that has been reported for NF-κB transcription factors [14]. Although the Meme Suite did not predict a comparable NF-κB binding site in the human *Ripk3* promoter, our lab has shown that there is high sequence conservation of the *Ripk3* promoter around the -1.0 kb and -0.2 kb regions across species [6]. This conservation guided our analysis of these regions in our ChIP experiments. Please note that because we could not pinpoint specific NFκB1 or IKBKG binding sequences in the human *Ripk3* promoter, we could not perform the relevant luciferase assays suggested above in HUVECs.

**Figure R1: Predicted NF-κB binding motif in the murine *Ripk3* promoter.** A position weight matrix of the NF-κB consensus binding motif was generated for the murine *Ripk3* (*mRipk3*) promoter using the MEME Suite (<https://meme-suite.org/meme/>). A binding site was predicted approximately 1.2 kb upstream of the transcription start site. Although a comparable binding motif was not predicted for the human *Ripk3* promoter with this software, our lab has shown high conservation of the *Ripk3* promoter between mice and humans around the -1.0 kb and -0.2 kb regions, as described above.

3. *(Major) “It would be helpful if there was additional validation of the GLoPro technique beyond just NFκB1.”*

Please see our response to Reviewer 2, question #4.

4. *(Major) “Does NFκB1 knock-down in MS1 cells affect RIPK3 expression?”*

We used the immortalized MS1 endothelial cell line to perform GLoPro because this assay requires clonal selection and a large number of cells (~4X10^8^), which are impractical hurdles when using primary endothelial cells like HUVECs. When we obtained our GLoPro results, we initially began validating them in the MS1 line. Indeed, we did see a small but significant decrease in *Ripk3* transcripts when we knocked down NFκB1 (see Figure R2A below). We likewise saw a small but significant decrease in luciferase activity when we knocked down NFκB1 in MS1 cells transfected with a luciferase reporter driven by a portion of the *Ripk3* promoter to which we can ChIP NFκB1 (see Figure R2B,D below). Finally, we saw a small but significant increase in *Ripk3* transcripts when we overexpressed NFκB1 in MS1 cells (see Figure R2C below). Together these data indicate that NFκB1 manipulation can impact *Ripk3* transcription in MS1 cells, but because this is an immortalized cell line, we thought it would be important to validate the ability of NFκB1 to influence *Ripk3* transcription in primary endothelial cells such as HUVECs (see response to Reviewer #1, question #5). Our HUVEC data do validate NFκB1 as a transcriptional regulator of *Ripk3*, but they reveal a different regulatory relationship from what we saw in MS1 cells: NFκB1 represses *Ripk3* transcription when HUVECs are challenged with TNFα—a major stimulus for NF-κB signaling. We believe the large and highly significant effects we see on *Ripk3* transcription and on RIPK3-mediated cell death in HUVECs following NFκB1 knockdown and TNFα stimulation are more compelling than the nominal changes we saw in baseline MS1 cells with NFκB1 manipulation.

**Figure R2: NFκB1 manipulation alters *Ripk3* transcription in the immortalized MS1 EC line.** (A) MS1 ECs were transduced for 72 hr with lentiviruses encoding scrambled shRNA (negative control) or shRNA against NFκB1. Transcripts of *Ripk3* were measured by qPCR. Data from 4 independent experiments were combined and are presented as relative fold change compared to normalized data from the negative control scrambled shRNA (dotted line). Error bars represent SD. Statistics were calculated using a one-sample, two-tailed *t* test. (B) Luciferase assays were performed in MS1 ECs co-transfected with a reporter construct encompassing 2281 bp of the *Ripk3* locus (including all three sites targeted by the *Ripk3* sgRNAs used in the GLoPro experiment) and with a constitutive Renilla luciferase control plasmid for 48 hr. Before transfection, cells were transduced with either a scrambled control shRNA or with an NFκB1-targeting shRNA. Ratios of relative luciferase:renilla activity were normalized to results from the scrambled control samples. Error bars represent SD of results from three independent experiments (with duplicate samples). Statistics were calculated using a two-tailed Student’s *t* test. (C) MS1 ECs were transfected with control or NFκB1 expression plasmids for 72 hr. Transcripts of *Ripk3* were measured by qPCR. Data from 3 independent experiments were combined. Statistics were calculated using a two-tailed Student’s *t* test. (D) ChIP assays were performed in MS1 ECs using antibodies against NFκB1 or against rabbit IgG (as a negative control). Immunoprecipitated (IP) DNA was isolated and amplified by PCR to determine whether NFκB1 bound the *Ripk3* promoter in four different locations. Input DNA was isolated from chromatin prior to IP and was used to confirm PCR efficiency. H_2_O was amplified with each primer set as a negative control.

In summary, we acknowledge the different effects on *Ripk3* transcription when we knockdown NFκB1 in MS1 cells (small reduction) versus HUVECs (big increase when cells are stimulated with TNFα). We also acknowledge that baseline RIPK3 protein is much more highly expressed in the two immortalized endothelial cell lines we analyzed (MS1 and C166) than in HUVECs (original Figure S1). We don’t know the reason for this expression difference, but we suspect the high levels of RIPK3 in MS1 cells made it beneficial for using GLoPro to identify proteins that sit at the *Ripk3* promoter and impact its transcription. Nevertheless, we maintain that functional validation of the true regulatory roles of our GLoPro-identified proteins is best reserved for primary endothelial cells like HUVECs with more physiological RIPK3 expression.*

*We have only seen substantial in vivo endothelial RIPK3 expression in mouse embryos during stages of active angiogenesis [1, 6, 7].

5. *(Minor) “It isn’t clear how the control probe was designed.”*

The control probe for the GLoPro analysis (non-targeting gRNA; NT-gRNA) was designed so as not to recognize any sequence in the mouse genome, as described [15].

6. *(Minor) “Are the GO terms shown in the figures complete or selected GO terms? This should be indicated in the figure legends.”*

The GO terms associated with the GLoPro heat map (original Figure 2; revised Figure S2A) were filtered to include terms that include “nuclear as cellular component” and “transcription as molecular function,” since our goal was to identify transcriptional regulators of *Ripk3*. The GO terms shown in the original Figure 3 (revised Figure S2A) are further (unfiltered) functional annotations of the 41 “nuclear” proteins identified in Figure 2. These distinctions are explained in the legend for Figure S2.

7. *(Minor) “Information on the number of replicates that were performed should be included in the figure legends.”*

This information is now included in the figure legends.

8. *(Minor) “In Figure 2, it would be helpful to show the data from the control oligo in the heat map for comparison purposes.”*

We have modified the heat map (now shown in revised Figure 1C) to accommodate this suggestion.

In summary, we have made the following additions and changes to this manuscript in response to the Reviewers’ suggestions:

- Combined original Figures 1 and 2
- Moved original Figure 3 to the supplement (now Figure S2)
- Generated new analysis of an additional GLoPro target protein and NF-κB signaling pathway component: IKBKG. We showed by immunostaining that IKBKG has significant nuclear localization in HUVECs grown under basal conditions or with TNFα stimulation (new Figure 3A). We also showed knockdown of IKBKG in HUVECs stimulated with TNFα significantly elevates *Ripk3* transcripts (new Figure 3B)*.

*Please note that while analyzing *Ripk3* transcription following IKBKG knockdown, we also repeated NFκB1 knockdown in the same experimental runs (both as a control and as further validation of the NFκB1 effects on *Ripk3* transcription from a different author). Our revised Figure 2A now includes our most current analyses of NFκB1 knockdown effects on *Ripk3* transcription. Note, the data from non-specific-siRNA-treated cells are identical for Figure 2A and 3B, since the analyses were performed at the same time (this is indicated in our new figure legend for Figure 3). Therefore, the effects of NFκB1 and IKBKG knockdown can be directly compared because they were generated in the same experiments, but we separated out the data into Figures 2A and 3B so that we could present the NFκB1 and IKBKG stories sequentially and logically.

- Generated all new ChIP-qPCR analysis of NFκB1 and IKBKG binding to the *Ripk3* promoter in HUVECs, both at basal conditions and after TNFα treatment (revised Figure 4). ChIP experiments were performed three separate times so that appropriate quantification and statistical analyses of the findings could be presented, as requested.
- Manuscript text was modified to include the new IKBKG data and to incorporate Reviewer suggestions.

Thank you for considering these revisions; we believe they shed additional novel insights into endothelial *Ripk3* transcriptional regulation, particularly under inflammatory stimulation.

Sincerely,

Courtney Griffin, Ph.D.

References for Rebuttal Letter

1.	Gao S, Griffin CT. RIPK3 modulates growth factor receptor expression in endothelial cells to support angiogenesis. *Angiogenesis.* 2021. Epub 2021/01/16. doi: 10.1007/s10456-020-09763-5. PubMed PMID: 33449298.

2.	Jacobsen MD, Weil M, Raff MC. Role of Ced-3/ICE-family proteases in staurosporine-induced programmed cell death. *J Cell Biol.* 1996;133(5):1041-51. Epub 1996/06/01. doi: 10.1083/jcb.133.5.1041. PubMed PMID: 8655577; PubMed Central PMCID: PMCPMC2120856.

3.	Galluzzi L, Kepp O, Kroemer G. RIP kinases initiate programmed necrosis. *J Mol Cell Biol.* 2009;1(1):8-10. Epub 2009/08/15. doi: 10.1093/jmcb/mjp007. PubMed PMID: 19679643.

4.	Narayan N, Lee IH, Borenstein R, Sun J, Wong R, Tong G, et al. The NAD-dependent deacetylase SIRT2 is required for programmed necrosis. *Nature.* 2012;492(7428):199-204. Epub 2012/12/04. doi: 10.1038/nature11700. PubMed PMID: 23201684.

5.	Degterev A, Zhou W, Maki JL, Yuan J. Assays for necroptosis and activity of RIP kinases. *Methods Enzymol.* 2014;545:1-33. doi: 10.1016/B978-0-12-801430-1.00001-9. PubMed PMID: 25065884.

6.	Colijn S, Gao S, Ingram KG, Menendez M, Muthukumar V, Silasi-Mansat R, et al. The NuRD chromatin-remodeling complex enzyme CHD4 prevents hypoxia-induced endothelial Ripk3 transcription and murine embryonic vascular rupture. *Cell Death Differ.* 2020;27(2):618-31. Epub 2019/06/27. doi: 10.1038/s41418-019-0376-8. PubMed PMID: 31235857.

7.	Colijn S, Muthukumar V, Xie J, Gao S, Griffin CT. Cell-specific and athero-protective roles for RIPK3 in a murine model of atherosclerosis. *Dis Model Mech.* 2020;13(1). Epub 2020/01/19. doi: 10.1242/dmm.041962. PubMed PMID: 31953345; PubMed Central PMCID: PMCPMC6994951.

8.	Lacorre DA, Baekkevold ES, Garrido I, Brandtzaeg P, Haraldsen G, Amalric F, et al. Plasticity of endothelial cells: rapid dedifferentiation of freshly isolated high endothelial venule endothelial cells outside the lymphoid tissue microenvironment. *Blood.* 2004;103(11):4164-72. Epub 2004/02/21. doi: 10.1182/blood-2003-10-3537. PubMed PMID: 14976058.

9.	Xia P, Gamble JR, Rye KA, Wang L, Hii CS, Cockerill P, et al. Tumor necrosis factor-alpha induces adhesion molecule expression through the sphingosine kinase pathway. *Proc Natl Acad Sci U S A.* 1998;95(24):14196-201. Epub 1998/11/25. doi: 10.1073/pnas.95.24.14196. PubMed PMID: 9826677; PubMed Central PMCID: PMCPMC24350.

10.	Israel A. The IKK complex, a central regulator of NF-kappaB activation. *Cold Spring Harb Perspect Biol.* 2010;2(3):a000158. Epub 2010/03/20. doi: 10.1101/cshperspect.a000158. PubMed PMID: 20300203; PubMed Central PMCID: PMCPMC2829958.

11.	Legarda-Addison D, Hase H, O'Donnell MA, Ting AT. NEMO/IKKgamma regulates an early NF-kappaB-independent cell-death checkpoint during TNF signaling. *Cell Death Differ.* 2009;16(9):1279-88. Epub 2009/04/18. doi: 10.1038/cdd.2009.41. PubMed PMID: 19373245; PubMed Central PMCID: PMCPMC2728158.

12.	Hwang B, McCool K, Wan J, Wuerzberger-Davis SM, Young EW, Choi EY, et al. IPO3-mediated Nonclassical Nuclear Import of NF-kappaB Essential Modulator (NEMO) Drives DNA Damage-dependent NF-kappaB Activation. *J Biol Chem.* 2015;290(29):17967-84. Epub 2015/06/11. doi: 10.1074/jbc.M115.645960. PubMed PMID: 26060253; PubMed Central PMCID: PMCPMC4505044.

13.	Myers SA, Wright J, Peckner R, Kalish BT, Zhang F, Carr SA. Discovery of proteins associated with a predefined genomic locus via dCas9-APEX-mediated proximity labeling. *Nat Methods.* 2018;15(6):437-9. Epub 2018/05/08. doi: 10.1038/s41592-018-0007-1. PubMed PMID: 29735997; PubMed Central PMCID: PMCPMC6202184.

14.	Wan F, Lenardo MJ. Specification of DNA binding activity of NF-kappaB proteins. *Cold Spring Harb Perspect Biol.* 2009;1(4):a000067. Epub 2010/01/13. doi: 10.1101/cshperspect.a000067. PubMed PMID: 20066093; PubMed Central PMCID: PMCPMC2773628.

15.	Doench JG, Fusi N, Sullender M, Hegde M, Vaimberg EW, Donovan KF, et al. Optimized sgRNA design to maximize activity and minimize off-target effects of CRISPR-Cas9. *Nat Biotechnol.* 2016;34(2):184-91. Epub 2016/01/19. doi: 10.1038/nbt.3437. PubMed PMID: 26780180; PubMed Central PMCID: PMCPMC4744125.
