# Supplementary material for: Genomic locus proteomic screening identifies the NF-κB signaling pathway components NFκB1 and IKBKG as transcriptional regulators of Ripk3 in endothelial cells
Source: PLoS One. 2021 Jun 21;16(6):e0253519. doi: 10.1371/journal.pone.0253519 (PMC8216549; doi:10.1371/journal.pone.0253519)
Supplement: Attachment — Submitted filename: Response to Reviewers PONE-D-2-35540R1.docx [file pone.0253519.s015.docx]

**Courtney T. Griffin, Ph.D.**

*Member, Cardiovascular Biology Research Program*

*Scott Zarrow Chair in Biomedical Research*

*Scientific Director, Oklahoma Center for Adult Stem Cell Research*

*President-elect, North American Vascular Biology Organization*

Oklahoma Medical Research Foundation

825 Northeast 13^th^ Street

Oklahoma City, OK 73104-5046

Telephone: 405-271-7073

Fax: 405-271-3137

Email: Courtney-Griffin@omrf.org

May 30, 2021

Ramani Ramchandran

Academic Editor

PLOS ONE

RE: Resubmission of PONE-D-20-35540R1

Dear Dr. Ramchandran:

Thank you for the recent reviews of our revised manuscript *“Genomic locus proteomic screening identifies the NF-κB signaling pathway components NFκB1 and IKBKG as transcriptional regulators of Ripk3 in endothelial cells.”* Reviewer #2 requested a more thorough description of our statistical methods; we appreciated this suggestion and have included responses below:

**Reviewer #2:**

*Minor comments: Although, authors have added statistical analysis information in the figures as well as legends, but it appears vague. Is there any rational to use two different statistics tests for example?*

*Figure 2: Error bars represent S.D. All statistics were calculated using a one-sample t and Wilcoxon test. (*) indicates statistical significance (p<0.05).*

We chose to use a one-sample t and Wilcoxon test to determine if the mean of ChIP enrichment (for NFκB1 or for IKBKG) is statistically different from the normalized negative IgG control. This test was chosen because we were comparing one variable: each mean enrichment value (independently) against the calculated IgG control enrichment levels.

*Figure 3: All quantified data are presented as mean S.D. all statistics were calculated using a two-way ANOVA with a Tukey’s multiple comparisons test. (*) indicates statistical significance (p<0.05).*

We chose to use a two-way ANOVA with a Tukey post hoc test because we were comparing two factors simultaneously: siRNAs (nonspecific or gene-specific) and treatment (vehicle or TNFα or Z-VAD-FMK or NSA).

*Author should add detail statistical analysis methods information for each panel and how they were compared in “Statistics” section.*

We have updated the “Statistics” section within the “Materials and methods” to say the following (new lines 259-268):

Results are presented as mean ± S.D. of *n* independent experiments (*n* is reported in the figure legends). Data normality was determined using the D'Agostino-Pearson omnibus test. Two-way ANOVA with a Tukey post hoc test was used to determine changes in HUVECs knocked down with nonspecific siRNA or gene of interest-specific siRNA after treatment with vehicle or TNFα or Z-VAD-FMK or NSA (Figs 2A, 2C, 2D, 3B). One-sample *t* and Wilcoxon test was used to determine fold-change of ChIP enrichment compared to the normalized negative IgG control (Figs 4A and 4B). Significance was determined by a *P*-value of 0.05 or less. All statistical analyses were achieved using GraphPad Prism8 software.

Thank you for this opportunity to improve the statistical descriptions associated with this revised manuscript.

Sincerely,

Courtney Griffin, Ph.D.
